# The value of pre-symptomatic genetic risk assessment for age-related macular degeneration: the Moran AMD Genetic Testing Assessment (MAGENTA) study—a study protocol for a randomized controlled trial

**DOI:** 10.1186/s13063-023-07436-4

**Published:** 2023-06-19

**Authors:** Emmanuel K. Addo, M. Elizabeth Hartnett, Paul S. Bernstein

**Affiliations:** 1 Department of Ophthalmology and Visual Sciences, John A. Moran Eye Center, University of Utah, 65 Mario Capecchi Drive, Salt Lake City, UT 84132 USA; 2 Department of Nutrition and Integrative Physiology, University of Utah, Salt Lake City, UT USA; 3 Department of Ophthalmology and Visual Sciences, Byers Eye Institute, Stanford University, Palo Alto, CA USA

**Keywords:** Carotenoids, Age-related macular degeneration, Genetic testing, Immediate and deferred disclosure, Lutein and zeaxanthin, Lifestyle modification, Pre-symptomatic, Randomized controlled trial

## Abstract

**Background:**

Age-related macular degeneration (AMD) is an irreversible blinding eye condition with complex genetic and environmental etiologies. Genetic testing for AMD for previously identified multiple-risk single nucleotide polymorphisms can help determine an individual’s future susceptibility. However, such testing has been discouraged until evidence shows that providing such information to symptomatic or pre-symptomatic individuals will alter their disease course. Therefore, we designed this study to investigate whether knowledge of AMD risk could stimulate the adoption of a healthier lifestyle that could lower the incidence of AMD later in life. We hypothesize that pre-symptomatic individuals informed of a high genetic risk of AMD are more likely to make quantifiable, positive lifestyle changes relative to participants informed of lower genetic risk or randomized to deferred disclosure of genetic testing results.

**Methods:**

The Moran AMD Genetic Testing Assessment (MAGENTA) study is a phase 2, single-center, prospective, double-masked, randomized controlled trial conducted at the John A. Moran Eye Center, University of Utah, Salt Lake City, Utah, USA. Participants are randomized by a 3:1 allocation ratio to immediate and deferred disclosure groups and followed for 12 months. Skin, ocular, and serum carotenoid status, as well as nutritional and social surveys, are assessed at study visits. Skin carotenoid assessment is by resonance Raman spectroscopy and reflectance spectroscopy, ocular carotenoids are measured with Heidelberg Spectralis autofluorescence imaging and fluorescence lifetime imaging ophthalmoscopy (FLIO), and serum carotenoids are quantified using high-performance liquid chromatography. The primary outcome evaluates changes in skin carotenoid status in response to genetic risk disclosure. The secondary outcomes examine changes in ocular and serum carotenoid status in response to genetic risk disclosure. Also, we will correlate AMD genetic risk with baseline ocular and systemic carotenoid status and FLIO.

**Discussion:**

MAGENTA will provide much-needed evidence on whether pre-symptomatic testing for AMD risk can lead to quantifiable long-term changes in behavior and lifestyle associated with a lower incidence of AMD later in life. Findings from the MAGENTA trial will facilitate the design of a future larger, longer-term, multicenter phase 3 trial that could feature subgroup analysis, expanded measures of lifestyle modification, and potential active nutritional interventions.

**Trial registration:**

ClinicalTrials.gov NCT05265624. Registered on March 3, 2022.

## Administrative information

The numbers in curly brackets in this protocol refer to the SPIRIT checklist item numbers. The order of the items has been modified to group similar items (see http://www.equator-network.org/reporting-guidelines/spirit-2013-statement-defining-standard-protocol-items-for-clinical-trials/).

**Table:** 

Title {1}	The Value of Pre-symptomatic Genetic Risk Assessment for Age-Related Macular Degeneration: The Moran AMD Genetic Testing Assessment Study (MAGENTA) – A Study Protocol for a Randomized Controlled Trial
Trial registration {2a and 2b}	ClinicalTrials.gov identifier: NCT05265624 Registered on March 3, 2022.
Protocol version {3}	IRB_00150093/ February 8, 2023
Funding {4}	NIH Grant EY033579
Author details {5a}	^1^Department of Ophthalmology, John A. Moran Eye Center, University of Utah Health, Salt Lake City, Utah, USA ^2^ Department of Nutrition and Integrative Physiology, University of Utah, Salt Lake City, Utah, USA ^3^ Department of Ophthalmology and Visual Sciences, Byers Eye Institute, Stanford University, Palo Alto, California, USA.
Name and contact information for the trial sponsor {5b}	National Institutes of Health (NIH), 9000 Rockville Pike, Bethesda, Maryland 20,892, USA
Role of sponsor {5c}	The funding sources have no role in the study design; collection, analysis, and interpretation of data; writing of the report; or the decision to submit the report for publication.

## Introduction

### Background and rationale {6a}

Age-related macular degeneration (AMD) is the leading cause of irreversible central vision loss among the elderly (> 55 years) in developed countries [1–3]. Currently, the exact pathogenesis remains to be elucidated, and with the increased life expectancy, it is estimated that about 288 million individuals worldwide will have AMD by 2040 [3]. Although there is no cure for AMD yet, early detection and treatment (i.e., lifestyle changes, nutritional supplements, and intravitreal injection) can delay the disease onset/progression and preserve vision. Modifiable (i.e., smoking, diet, and sunlight exposure) and non-modifiable (i.e., aging and genetic susceptibility) risk factors have been implicated in the etiology of the disease [4].

In recent years, research has demonstrated that variants in the genes, complement factor H (*CFH*), age-related maculopathy susceptibility protein 2 (*ARMS2*)/high-temperature requirement factor A1 (*HTRA1*), and other genetic loci are significant risk factors for AMD [5–7] and that nutritional supplementation with age-related eye disease study 2 (AREDS2) antioxidant vitamins, minerals, and carotenoids could slow down the progression of this blinding disorder [8]. Despite recommendations by the American Academy of Ophthalmology (AAO) against routine genetic testing for AMD risk, a considerable number of individuals express interest in pursuing such testing, and direct-to-consumer laboratories already market them commercially. The AAO’s expert panel discouraged the clinical use of such testing, not because they are unreliable at predicting the risk of eventual visual loss from AMD, but rather because there is no proof yet that providing such knowledge to symptomatic or pre-symptomatic individuals will alter the course of the disease [9, 10].

Healthy behaviors such as smoking cessation, maintaining a healthy weight, decreased light exposure, and increased ingestion of diets rich in carotenoids are associated with delayed AMD incidence and progression [8, 11–14]. These changes in lifestyle all result in increased systemic and ocular levels of lutein (L), zeaxanthin (Z), and other carotenoids. These dietary xanthophyll carotenoids (L and Z) uniquely deposit in the human eye, where they are collectively known as macular pigment (MP) [15, 16]. MP functions as a light screener to filter out energetic blue light that can damage photoreceptors and acts as a localized depot of antioxidants to mitigate light-induced oxidative stress [17–19]. Recent studies report an inverse relationship between the ingestion of diets rich in dark green leafy vegetables and orange/yellow fruits and vegetables and the risk of progression to advanced AMD [20, 21]. Furthermore, studies demonstrate that changes in systemic and ocular biomarkers of carotenoid status and risk of AMD can be reproducibly and reliably measured using validated, non-invasive, and objective devices [22, 23].

A prospective study to show that knowledge of AMD risk could decrease the incidence of AMD decades later would settle this controversy and would be consistent with research recommendations of the AAO expert panel, but it is not currently feasible due to the large number of subjects and the prolonged time required. Instead, we propose a shorter, phase 2 randomized clinical trial to determine if quantifiable biomarkers of healthy behavior (skin and ocular carotenoid levels) improve in response to knowledge of AMD risk. We speculate that compared to subjects who have deferred disclosure or who are informed of low risk, individuals informed of a high risk of eventual AMD will be more likely to make sustained changes in behavior associated with decreased incidence of AMD later in life. The findings of this study will then be used to design a future phase 3 multicenter trial.

### Objectives {7}

We hypothesize that individuals informed of a high genetic risk of eventual progression to advanced AMD are more likely to make quantifiable, positive lifestyle changes than participants informed of lower genetic risk or randomized to deferred risk disclosure. Thus, our primary aim for this study will investigate changes in subjects’ skin carotenoid status using resonance Raman spectroscopy (RRS) and reflection spectroscopy (RS). Our secondary aim will determine changes in participants’ macular pigment and serum carotenoid status in response to AMD genetic risk disclosure. Our exploratory aim will correlate participants’ AMD genetic risk profiles with baseline ocular and systemic carotenoid status and fluorescence lifetime imaging ophthalmoscopy (FLIO). This will provide information on the influence of inherited factors on xanthophyll carotenoid metabolism and macular deposition and determine whether lifestyle modifications alter the AMD-associated FLIO patterns observed in the long spectral channel in up to 1/3 of clinically normal middle-aged individuals.

### Trial design {8}

**Fig. 1:**
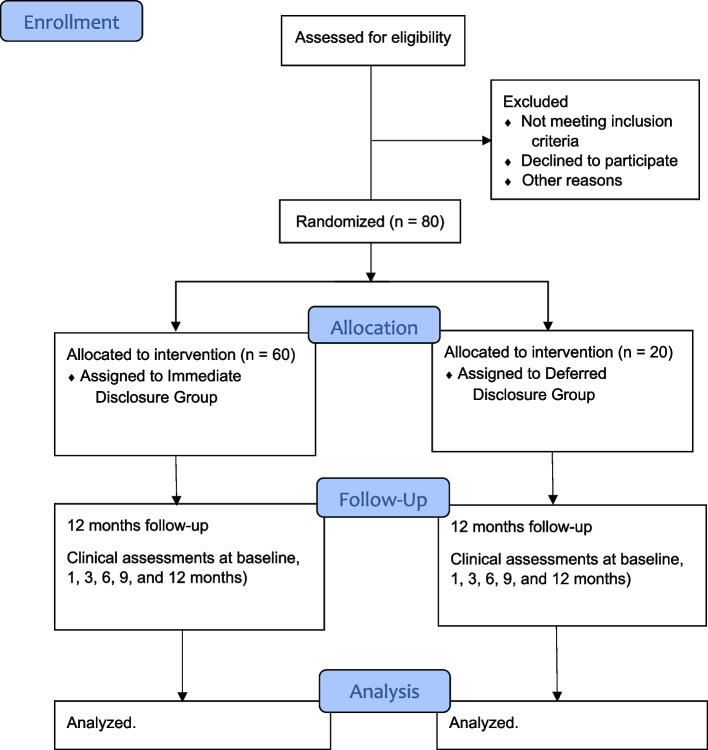
Moran AMD Genetic Testing Assessment (MAGENTA) study consolidated statement of reporting trials flow diagram

The Moran AMD Genetic Testing Assessment (MAGENTA) study is a phase 2, single-center, prospective, double-masked, randomized controlled trial conducted at the John A. Moran Eye Center, University of Utah, Salt Lake City, Utah, USA. The study will span a 2-year period. Participants will be randomly assigned by a 3:1 allocation ratio to the immediate disclosure or deferred disclosure of AMD genetic risk groups but will not be informed of the study assignment until the genetic risk report is returned from the testing laboratory (approximately 1 month later). Deferred disclosure will occur at the 12-month visit. Each participant will have one eye designated as the study eye for purposes of macular pigment and FLIO measurements (typically the non-dominant eye unless the physician or participant decides otherwise). The flowchart in Fig. 1 illustrates participant enrollment, random assignment, follow-up, and analysis for the MAGENTA trial.

## Methods: participants, interventions, and outcomes

### Study setting {9}

MAGENTA is a single-center study conducted at the John A. Moran Eye Center, the University of Utah, Salt Lake City, Utah, USA. Study subjects will be primarily recruited from clinics at the Moran Eye Center, satellite clinics, and the university community at large. All study visits will be at the Moran Eye Center.

## Eligibility criteria {10}

### Inclusion criteria

*The inclusion criteria are as follows:*

1. Participants must be between 18 and 64 years of age.
2. Participants must be Caucasian, as the genetic test is only validated in Caucasians.
3. Participants can have a positive family history of AMD.

### Exclusion criteria

*Participants will be excluded if they:*

1. Have personal history of AMD or have had prior genetic testing for AMD risk.
2. Are non-Caucasian.
3. Are employees of the Moran or other eye care practice (likely to have more knowledge about AMD than a layperson).
4. Anticipate having cataract surgery in the upcoming year, as this can affect macular pigment measurement.
5. Have significant eye disease associated with macular pigment abnormalities such as Stargardt disease, albinism, or macular telangiectasia type II.
6. Have a major psychiatric disorder.

### Who will take informed consent? {26a}

All participants will be required to provide written informed consent in compliance with International Conference on Harmonization (ICH) Guidance E6 before study enrollment. The Institutional Review Board (IRB) of the University of Utah, Salt Lake City, Utah, granted ethical approval for this study. MAGENTA will adhere to the tenets of the Declaration of Helsinki, the ICH Harmonized Tripartite Guidance for Good Clinical Practice (IHC-GCP E6 [R1]) and will comply with the code of ethics regarding participant enrollment, study assessment, and data protection.

A trained study staff member will obtain written informed consent from each participant. The study team will communicate the study’s purpose, the assessment procedures involved, and the potential hazards to the participants in non-technical terms. The participant will have ample time to consider the study’s implications before deciding to participate in the study. The participant will be required to sign and date an informed consent form (ICF) and may opt to withdraw from the study at any time without jeopardizing their medical care. The co-principal investigators (co-PIs) will retain the original, signed ICF for study participation in the subject’s medical record and will provide the participant with a copy of the signed consent form.

Importantly, if there are any amendments to the approved protocol which may directly affect the subject’s decision to continue participation in the study, the ICF will be revised to integrate the changes to the protocol, and the participant will be required to endorse the IRB-approved amended ICF.

### Additional consent provisions for collection and use of participant data and biological specimens {26b}

The consent form will request participants’ authorization to disclose pertinent data to regulatory authorities and the IRB, as deemed appropriate by the research team. There will be no collection and storage of biological samples for ancillary studies in the MAGENTA trial.

## Interventions

### Explanation for the choice of comparators {6b}

MAGENTA is a phase 2, single-center, prospective, randomized, controlled clinical trial comparing the effect of immediate versus deferred disclosure of AMD genetic risk on biomarkers of ocular and systemic carotenoid status. Thus, an early or late disclosure will help determine the impact of an AMD genetic risk test on an individual’s decision to adopt a healthy lifestyle.

### Intervention description {11a}

After retinal images are obtained and reviewed by the study co-PIs, eligible participants who provide informed written consent will be randomized in a ratio of 3:1 to the two study arms (the immediate or the deferred disclosure group). The study team will instruct participants in both study groups to carry out their normal daily routine following study assignments.

Baseline measurements of each participant’s carotenoid status (in the skin, serum, and eye) and nutritional and behavioral surveys will be performed at enrollment. A month afterward, participants will be assigned a study group following the availability of AMD genetic testing results. Repeated measurements of the participant’s systemic carotenoid status, macular pigment, and nutritional surveys will be carried out at subsequent study visits.

### Criteria for discontinuing or modifying allocated interventions {11b}

Although we expect and encourage enrolled participants to complete the study visits, participants are at liberty to withdraw from the study at any time for any reason. Also, the co-PIs reserve the right to withdraw a participant from the study in the event of an intercurrent illness, adverse event, non-compliance, or other reasons.

### Strategies to improve adherence to interventions {11c}

Study participants’ follow-up visits are scheduled at their convenience. Hence, prior to a follow-up appointment, the study coordinator contacts participants over the phone or through text messages to remind participants of their upcoming study visit. Also, participants receive reimbursement for volunteering their time in the form of a gift card. These measures ensure that participants comply with the study protocol.

### Relevant concomitant care permitted or prohibited during the trial {11d}

The participants will have no study-related dietary restrictions, as this trial is behavioral/social in its design. Hence, participants will consume carotenoids with their regular diet, and the LZQ™ quantitative food frequency questionnaire will assess participants’ carotenoid intake.

### Provisions for post-trial care {30}

All participants who complete the study (i.e., undergo a final assessment in the twelfth month) will have the opportunity to meet the psychological counselor for post-trial stress management.

### Outcomes {12}

This study’s primary outcome will determine change over 1 year in skin carotenoid status assessed by RRS and RS in response to genetic risk disclosure. We hypothesize that disclosure of high AMD risk will motivate subjects to make lifestyle changes that will improve the carotenoid levels in the subjects’ skin as assessed by resonance Raman spectroscopy and reflectance spectroscopy relative to lower risk disclosure or deferred disclosure. We will also compare disclosure of any AMD risk versus deferred disclosure.

The secondary outcome will investigate changes over 1 year in ocular and serum carotenoid status in response to genetic risk disclosure. We hypothesize that disclosure of high AMD risk will motivate subjects to make lifestyle changes that will improve the carotenoid levels in the subjects’ macula and serum as assessed by autofluorescence intensity and lifetime imaging (macula) and high-performance liquid chromatography (serum) relative to lower risk disclosure or deferred disclosure.

The exploratory outcome will correlate the various AMD risk alleles singly and in aggregate with our baseline measures of ocular and systemic carotenoid status to ascertain whether AMD-associated genetic risk factors influence carotenoid levels. We will also correlate our AMD genetic risk profiles with FLIO imaging to learn if there is a genetic basis for the AMD-associated FLIO patterns that we observe in the long spectral channel in up to 1/3 of clinically normal middle-aged individuals and to learn if lifestyle changes alter these patterns. In addition, we will evaluate the impact of genetic risk disclosure using standardized surveys to identify participants who may benefit from psychological counseling.

### Participant timeline {13}

MAGENTA is expected to last for two years, depending on the pace of enrollment. Table 1 provides details of participants’ study timelines and assessments.

**Table 1:** Timeline and assessment schedule for MAGENTA trial

Visit	Screening/baseline	Disclosure	Monitoring			Closeout
Month	0	1	3	6	9	12
Informed consent	X					
Counseling on AMD risk factors and reduction strategies	X	X				
Blood or saliva collection for genetic testing	X					
Color fundus photography and OCT to confirm eligibility	X					
Randomization		X				
Disclosure (or deferral) of genetic testing results		X				X
Skin carotenoid levels by RRS and RS	X	X	X	X	X	X
Ocular carotenoid levels by AFI and FLIO	X			X		X
Nutritional and social surveys	X		X	X	X	X
Serum carotenoid levels	X			X		X
Height and weight measurement for BMI	X			X		X

*AMD* age-related macular degeneration, *OCT* optical coherence tomography, *RRS* resonance Raman spectroscopy, *RS* reflectance spectroscopy, *AFI* autofluorescence imaging, *FLIO* fluorescence lifetime imaging ophthalmoscopy, *BMI* body mass index

### Sample size {14}

Our power calculation for the primary comparisons utilizes the pilot study data. We enrolled 16 subjects under age 60 interested in learning their genetic risk of AMD and randomized them 3:1 to immediate and deferred disclosure. Although this pilot study was underpowered to draw any definitive conclusions, we observed a trend that early disclosure of any degree of risk for AMD was associated with an increase in skin carotenoid status relative to the deferred disclosure group whose skin carotenoids scores remained essentially unchanged. We found that with 60 patients (after 25% drop-out from 80 enrolled patients) and an allocation ratio of 3:1 to early and late disclosure groups, there is 80% power to detect a 40.5% difference for our primary comparison. This compares favorably with the > 100% changes achievable with diet alone.

### Recruitment {15}

Participant recruitment for the MAGENTA study will be primarily from the John A. Moran Eye Center, University of Utah, Salt Lake City, UT, USA. We will reach out to participants who attend in-person visits to the study site or who are referred by family members or by treating physicians. We will also use written or electronic record review, written advertising (flyers, brochures, website postings, newspaper ads, etc.) and a database for which participants have given prior permission to be contacted for research studies. Potential and interested volunteers will be referred to the study coordinator who will establish contact with them to explain the study and determine if the individual meets initial eligibility requirements. Participants who agree to participate in the study will complete a telephone-based screening to ensure they meet inclusion criteria. If eligible, individuals will be asked to schedule an in-person baseline visit. An authorized member of the study staff will obtain written informed consent to the IRB-approved study protocol before enrollment and study assessments. Consultant ophthalmologists with retinal subspecialty will review baseline data, including color fundus photography and optical coherence tomography to confirm study eligibility. Once eligibility is confirmed, participants will schedule subsequent study visits with the study coordinator.

## Assignment of interventions: allocation

### Sequence generation {16a}

The participants will be randomly assigned in a 3:1 ratio using computer-generated random sequences and block stratification to either the immediate or deferred disclosure study groups. The essence of randomization is to guarantee an equal allocation of participants across all study arms.

### Concealment mechanism {16b}

**Fig. 2:**
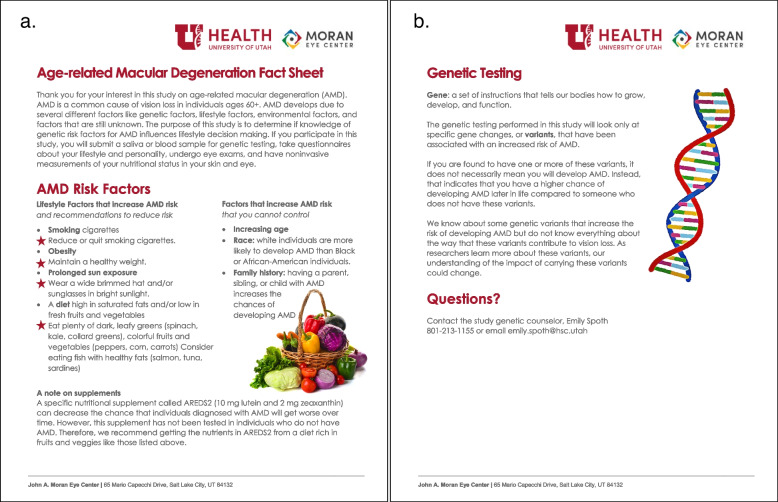
AMD fact sheet (**a**) and genetic testing handout (**b**) discussed with and given out to each participant at enrollment and randomization

We will obtain a randomization code list using computer-generated sequences. The random code list ensures that participants’ study allocation is concealed from both the participant and the investigators. Moreover, the licensed ophthalmic genetic counselor at the John A. Moran Eye Center who is not involved with the study assessments, will meet with participants to inform them of their disclosure assignment using the randomization code list, review the AMD risk factors and reduction strategies with participants in a standardized manner (Fig. 2), and will address any questions or concerns participants may have.

### Implementation {16c}

MAGENTA is a double-masked trial; hence, both researchers and study participants will be initially masked to the study assignment. A study statistician not involved in the data collection and who has no contact with the study participants generates the allocation assignment using a computerized random sequence generator. The masked study coordinator enrolls study participants following establishment of study eligibility, and the unmasked licensed ophthalmic genetic counselor assigns participants to the study intervention (i.e., the Immediate or Deferred Disclosure groups). By design, the subjects in the Immediate Disclosure group become unmasked at this point to their primary assignment group and AMD genetic risk, while the Deferred Disclosure group subjects become unmasked to their primary assignment group but remain masked to their AMD genetic risk.

## Assignment of interventions: blinding

### Who will be blinded? {17a}

The study participants, clinicians, and research staff involved in all participant evaluations will be masked to the study treatment assignments.

### Procedure for unblinding if needed {17b}

If a medical emergency arises and the treatment details of a participant are required, the co-PIs may unmask the treatment assignment for that specific participant and provide the necessary information to the relevant parties. However, if a participant’s treatment assignment has been unmasked due to an unexpected adverse event or medical emergency, they will no longer be able to continue participating in the study.

For regulatory reporting and if required by local regulations, the sponsor-investigator will unmask study treatment for all serious, unexpected adverse reactions related to the study.

## Data collection and management

### Plans for assessment and collection of outcomes {18a}

Once a participant is enrolled in the study, socio-demographic information, behavioral and social surveys, skin, serum, and ocular carotenoid status will be assessed at study visits. Trained study staff will perform the various study assessments and will enter the various information into the subject case report forms (CRF). The data collected will be entered into the Research Electronic Data Capture (REDCap) database maintained at the University of Utah with help from the Center for Clinical and Translational Science (CCTS). Data will be reviewed and checked regularly for quality and completeness using computerized and manual procedures. Access to the study database will be strictly authorized. The study assessments are outlined below.

### Demographic and lifestyle information

The demographic and lifestyle information of participants includes contact details (i.e., name, date of birth, phone number(s), place of residence, email address), education, ethnicity, occupation, height and weight (for body mass index [BMI] calculation), medical history, ocular medical history, smoking habits (history and frequency), and alcohol consumption (average intake per week and frequency).

### Dietary questionnaires and psychological/behavioral surveys

Participants will be required to complete nutritional and social surveys. Participants may choose to complete the surveys electronically or on paper either at their study visits or in advance of each visit. Two validated instruments, the Hospital Anxiety and Depression Scale (HADS) and the Impact of Events Scale (IES), will be used to evaluate the psychological impact of genetic risk communication and identify participants who may benefit from psychological counseling. Both scales are based on feelings and actions over the past week, and both have been used extensively in genetic counseling research [24].

The HADS is frequently used to measure the levels of anxiety and depression in non-psychiatric patients. The HADS is comprised of two distinct subscales, namely the HADS-A for anxiety and the HADS-D for depression. Both subscales contain seven questions, each rated on a scale of 0 to 3. A higher score on either subscale, ranging from 0 to 21, indicates an increased probability of experiencing distress. A score of 11 to 21 suggests the need for intervention [25]. The HADS has been demonstrated to possess good validity in non-psychiatric healthcare clinic settings [26].

The IES evaluates the distressing impact that knowledge of genetic risk may evoke [27]. This is a self-report measure comprising 15 items that evaluate subjective distress reactions, specifically intrusive thoughts, feelings, and imagery (7 items), as well as avoidance responses (8 items). Avoidance responses pertain to the avoidance of feelings, thoughts, or situations that might trigger memories of the event. Each question is rated on a scale of 0 (not at all) to 5 (often), with higher scores indicating greater distress. The total score ranges from 0 to 75, and a score of 26 or more indicates moderate to severe stress, thereby necessitating intervention. The IES has been demonstrated to possess good validity [28].

Moreover, participants will complete the LZQ™ quantitative food frequency questionnaire (analyzed by Elizabeth Johnson, Ph.D., at Tufts University, Boston, MA, USA) that captures ~ 90% of the lutein/zeaxanthin foods consumed in the USA, based on National Health and Nutrition Examination Survey (NHANES) data.

### Genetic testing for AMD risk

AMD risk will be assessed using methods established by Pappas et al. [29] based on an individual’s combination of genotypes at *CFH-CFHR5* and *ARMS2/HTRA1*, the two loci responsible for the vast majority of genetic AMD risk. For the *CFH-CFHR5* locus, rs800292, rs1061170, and rs12144939 will be used to determine risk, neutral, or protective haplotypes. At the *ARMS2/HTRA1* locus, the number of risk alleles at rs10490924 will assess risk. Diplotype combinations at these two loci will be ranked in order of increasing AMD odds ratio and partitioned into low-, intermediate-, and high-risk groups. Odds ratios from initial publication were refined through the inclusion of additional samples. Alongside odds ratios, 1000 Genomes Project (1000G) EUR frequencies will be used to balance our dual goals of accurate risk assessment and equally sized groups. Because relatives of individuals with AMD will be encouraged to enroll in the study, recruitment is assumed to be biased toward AMD risk genotypes. Accordingly, rather than designing risk groups to be equally sized according to 1000G frequencies, the low-risk group was designed to represent 50% of individuals, while roughly 25% of individuals will fall into the intermediate group and nearly 22% will be high risk (see Table 2). The remaining percentages will represent individuals with haplotypes that are too rare to accurately establish AMD risk.

**Table 2:** AMD risk assessment using combination of genotypes at *CFH-CFHR5* and *ARMS2/HTRA1*

*CFH-CFHR5* diplotype	*ARMS2/HTRA1* risk alleles	Odds ratio	1000G EUR Freq	Combined frequency	Relative risk
Prot/Prot	0	0.28	0.095	0.504	Low
Neut/Prot	0	0.34	0.156		
Prot/Prot	1	0.41	0.059		
Risk/Prot	0	0.43	0.166		
Neut/Neut	0	0.48	0.028		
Neut/Prot	1	0.7	0.055	0.251	Intermediate
Risk/Neut	0	0.86	0.073		
Risk/Prot	1	0.92	0.107		
Prot/Prot	2	1.17	0.006		
Neut/Neut	1	1.24	0.01		
Risk/Risk	0	1.53	0.099	0.215	High
Neut/Prot	2	1.9	0.006		
Risk/Neut	1	2.01	0.042		
Risk/Risk	1	3.93	0.05		
Risk/Prot	2	3.96	0.008		
Neut/Neut	2	5.25	0.002		
Risk/Neut	2	7.8	0.004		
Risk/Risk	2	12.6	0.004		

*Neut* neutral haplotype, *Prot* protective haplotype, *CFH-CFHR5* complement factor H-complement factor H-related 5, *ARMS2/HTRA1* age-related maculopathy susceptibility protein 2/high-temperature requirement factor A1

### Skin carotenoid measurement

#### Resonance Raman spectroscopy (RRS)

Skin carotenoid measurements will be assessed using a resonance Raman spectroscopy device. This validated non-invasive device serves as a biomarker of fruit and vegetable intake that significantly correlates with skin and serum total carotenoid levels [30–33]. MAGENTA uses a high-sensitivity scanner suitable for measurements of adult skin carotenoids. The Raman devices present little or no risk to the subjects. In summary, the measurements are taken by placing a probe on the subject’s palm with a 488-nm blue laser light that does not generate heat and is harmless to one’s vision as long as it is not viewed directly (similar in risk to a laser pointer). The device collects back-scattered light and has a holographic notch filter that rejects Rayleigh-scattered light. The Peltier-cooled spectrograph analyzes the resulting fluorescence and Raman-shifted light. The peak intensity represents the C = C vibration of carotenoids at about 1525 cm^−1^ (known as resonance Raman units [RRU]). The device is calibrated daily, and the measurements require 30 s of skin contact and another 30 s to obtain the reading. Overall, three measurements will be taken, and the average used for statistical analyses.

#### Reflection spectroscopy (RS)

We use the Veggie Meter® produced by Longevity Link Corporation, Salt Lake City, UT, USA, to assess skin carotenoid status by pressure-mediated reflectometry. The Veggie Meter® is a validated, rapid, non-invasive, and portable device that is a biomarker for fruit and vegetable intake, and its skin carotenoid score has a strong positive significant correlation with skin resonance Raman spectroscopy and serum total carotenoids [32, 34–36]. Briefly, the skin tissue of interest (i.e., the index finger) is illuminated with broad-band white light spanning the spectral range from 350 to 850 nm, and the spectral composition of the diffusively reflected light is analyzed in real-time. Participants gently press their index fingers against the convex lens surface with the help of a spring-loaded cover, which momentarily squeezes blood out of the illuminated tissue volume, reducing oxyhemoglobins and other chromophores’ influence on the reflection spectra. A laptop computer connected to the Veggie Meter® regulates the light exposure, data acquisition, processing, and display of the reflection spectra. The display provides the skin carotenoid score on a scale from 0 to 800 reflection units (RU). Three single readings will be taken, and the average score will be deemed the final skin carotenoid score. Before each measurement, participants will have their hands washed, and the device is calibrated daily with dark and white reference sticks.

### Macular pigment measurement

#### Pupillary dilation

Participants’ pupils will be dilated before carrying out macular pigment imaging. Thus, a drop each of 1% tropicamide and 2.5% phenylephrine will be used for dilation. These eye drops are considered standard-of-care for dilation in ophthalmological practices.

#### Participant MP assessment

Participants’ macular pigment will be measured using the dual-wavelength autofluorescence method on the Heidelberg Multicolor Spectralis (Heidelberg Engineering GmbH, Heidelberg, Germany), which measures the attenuation of lipofuscin autofluorescence by the blue absorbing macular pigment [37]. First, the examiner enters the participant’s details into the Heidelberg Eye Explorer (HEYEX version 1.7.1.0) software and asks the participant to fixate on a target with the dilated study eye. The fixation ensures good alignment and camera focus for quality retinal imaging.

Autofluorescence images will be collected as the macula is raster-scanned sequentially with alternating 486 nm (blue) and 518 nm (green) lasers. Macular pigment images are obtained by digitally subtracting the green image from the blue image using appropriate correction factors to compensate for the absorption spectrum of the macular carotenoid pigment and then analyzed using a beta version of Heidelberg’s proprietary macular pigment analysis software after setting the zero point at 9° eccentricity from the point of fixation. We used 9° eccentricity to enable consistency and comparison with previous studies and to avoid the optic nerve and retinal blood vessels’ effects on MP measurements. Subsequently, MP measurements at 0.5°, 2°, and 9° are recorded and used for statistical analysis. This instrument and software have proven highly reliable and reproducible [23, 38, 39], especially when measuring macular pigment optical volume at 9° eccentricity (MPOV 9°), which indicates the total of all MPOD values for all pixels with valid results within 9° eccentricity.

#### Fluorescence lifetime imaging ophthalmoscopy (FLIO)

FLIO is a non-invasive imaging modality based on fundus autofluorescence (FAF) intensity imaging constructed on a Heidelberg Retina Angiograph cSLO (HRA2, Heidelberg Engineering GmbH, Heidelberg, Germany). Thus, for every FLIO measurement, not only do we obtain FAF intensity images but also FAF lifetime (i.e., the timeframe from the excitation of the retinal fluorescence to the detection of the fluorescence signal). These FAF lifetimes provide unique ways to understand fluorophore’s metabolic environment [40]. Before FLIO imaging, the examiner ensures that participant details are entered correctly, the room is completely dark, the pupil is maximally dilated, and the participant is comfortably seated with eyes properly aligned with the internal fixation mark.

FLIO excites retinal fluorophores with a 473-nm wavelength pulsed diode laser-emitting pulses with a frequency of 80 MHz at full width at half maximum of 89 ps and records FAF lifetimes from a 30° retinal field (256 by 256 pixels) using the principle of time-correlated single photon counting [41].

According to the regulations outlined by the American National Standards Institute (ANSI) Z136.1–2000, the Heidelberg FLIO system meets all safety requirements for a class 1 laser, and its pulse energy is nearly 10^4^ times smaller than the acceptable exposure limit [42]. This ensures that the laser used by the FLIO system is safe and does not pose a risk of retinal damage to patients. Two hybrid photo-multipliers (HPM-100–40, Becker & Hickl GmbH, Berlin, Germany) detect fluorescence photons from each pixel in 1024 time channels, resulting in two separate spectral channels. The short spectral channel (SSC; 498–560 nm) and the long spectral channel (LSC; 560–720 nm) [43].

The FLIO system is equipped with a high-contrast confocal infrared reflectance (IR) image to track eye movements during measurements. This ensures that each FAF lifetime corresponds to the appropriate spatial retinal location. To obtain high-quality and reliable FLIO images, a minimum signal threshold of about 1000 photons per pixel is required, which takes approximately 2 min to measure per eye.

Commercial software (SPCImage 4.4.2; Becker & Hickl GmbH, Berlin, Germany) will be used to analyze fluorescence data. By calculating the least-square fit of a series of three exponential functions, the fluorescence decay will be approximated, and a 3 × 3 pixel binning will be used for noise reduction where necessary. The amplitude-weighted mean fluorescence decay time (τm), which represents the average of the 3 decay time constants from the fit, will then be used for further analysis [43–45].

#### Serum carotenoid concentrations extraction and quantification

Blood samples obtained from study participants will be analyzed for serum carotenoid concentrations using well-established laboratory protocols that provide baseline separations of all common dietary carotenoids [46, 47]. At each study visit, we will use 6 mL blood collection tubes (BD Vacutainer K_2_EDTA; Becton, Dickinson and Company, NJ, USA) to collect blood samples following standard techniques. The collection tubes are inverted a few times to ensure thorough mixing of the anticoagulant (K_2_EDTA) with the blood. The blood samples are allowed to sit at room temperature for 30 min and then centrifuged for 10 min at 3000 × *g* using a Sorvall™ ST 16 Centrifuge (ThermoFisher Scientific Inc., Waltham, MA, USA). After centrifugation, the serum (sample of interest) is separated from the whole blood, kept in a well-labeled storage tube, and stored at − 80^0^C until further analysis.

For serum carotenoid extraction, ethanol containing 0.1% butylated hydroxytoluene will be added to 200 μL of serum to separate the proteins and followed by ethyl acetate for carotenoid extraction. The sample is then vortexed for 30 s and centrifuged at 2000 × *g* for 5 min. Further extraction with ethyl acetate will be carried out twice and then once with hexane. All the organic extracts obtained will be dried down under nitrogen gas. Further, the organic extracts are cleaned with hexane/methanol/distilled water, and the supernatant is recovered and dried. The resulting residues will be resuspended in HPLC mobile phase (methanol: methyl tert-butyl ether [80:20, *v*/*v*]) and centrifuged at 2000 × *g* for 10 min. The supernatant will be analyzed using an Agilent 1260 series HPLC (Agilent Technologies Inc., Santa Clara, CA, USA) on a C30 column (YMC Carotenoids, Allentown, PA, USA; 25 cm length × 4.6 mm internal diameter; maintained at room temperature) using diode array detection at a wavelength of 450 nm and a flow rate of 1 mL/min for 50 min. Diode-array spectra and co-elution with authentic standards identify and confirm the various carotenoid peaks.

Standard solutions of each carotenoid of interest were prepared with known concentrations calculated spectroscopically using appropriate extinction coefficients and then injected in different volumes to achieve final injected amounts. Serum carotenoid concentrations will be quantified based on standard curves of known quantities of the authentic carotenoid standards injected into the HPLC system versus integrated ultraviolet/visible absorbance of their eluted peaks.

### Plans to promote participant retention and complete follow-up {18b}

To ensure study completion and participant retention, follow-up visits are scheduled in a manner that is convenient for study participants. Prior to these appointments, the study team will reach out to participants through various communication channels such as email, phone, or text message. Additionally, study participants will receive reimbursement in the form of a gift card to cover their time and transportation expenses.

### Data management {19}

All study data will be entered into REDCap and will be accessible to only authorized personnel. Further, the study monitoring team from the University of Utah Compliance Office will review the database for accuracy and completeness.

### Confidentiality {27}

The REDCap servers are equipped with encryption and password protection, as well as Health Insurance Portability and Accountability Act (HIPAA) compliance, making them accessible only to authorized study members. Additionally, hard copy data collection forms will be stored in a locked cabinet with restricted access, limited only to designated members. The statistical team will have access to a de-identified database for interim statistical analyses.

### Plans for collection, laboratory evaluation, and storage of biological specimens for genetic or molecular analysis in this trial/future use {33}

A study staff member trained in the protocol will carry out study assessments following standard procedures, and trained laboratory staff will process and analyze the blood samples following standard laboratory procedures. Participants’ identities will be coded so that samples cannot be traced to a specific person.

## Statistical methods

### Statistical methods for primary and secondary outcomes {20a}

Baseline characteristics between the two study groups will be assessed for statistically significant differences using descriptive statistics. Independent sample *t*-tests (mean, standard deviation) will be used for continuous variables, while chi-square tests will be used for categorical variables. We expect to have no statistically significant differences in baseline characteristics in both groups because of the randomization process. Nevertheless, any between-group differences in baseline characteristics will be controlled for in subsequent analyses, as appropriate.

Feasibility aim: We will monitor the proportion of participants who complete the monitoring and closeout visits by trial arm assignment and provide estimates and 95% confidence intervals for those rates.

Analytic aim: Our primary analysis will compare follow-up carotenoid level between the randomized early and late disclosure groups while accounting for AMD risk (high, medium, or low, denoted $RG_{\mathit l\mathit o\mathit w}$,$RG_{med}$,$RG_{\mathit h\mathit i}$) and baseline carotenoid level (denoted $BCL$) [48] under the longitudinal model:

$${Y}_{t}={\alpha +\beta }_{1}\mathrm{BCL}+{\beta }_{2t}{\mathrm{RG}}_{\mathrm{low}}\times {T}_{\mathrm{early}}+{\beta }_{3t}{\mathrm{RG}}_{\mathrm{low}}\times {T}_{\mathrm{late}}+{\beta }_{4t}{\mathrm{RG}}_{\mathrm{med}}\times {T}_{\mathrm{early}}+{\beta }_{5t}{\mathrm{RG}}_{\mathrm{med}}\times {T}_{\mathrm{late}}+{\beta }_{6t}{\mathrm{RG}}_{\mathrm{hi}}\times {T}_{\mathrm{early}}+{\beta }_{7t}{\mathrm{RG}}_{\mathrm{hi}}\times {T}_{\mathrm{late}}+{\epsilon }_{t}$$

Here ${Y}_{t}$ denotes the carotenoid level at time $t$, ${T}_{\mathrm{early}}$, and ${T}_{\mathrm{late}}$ are indicators for randomization to early and late disclosure, and ${\epsilon }_{t}$ is the residual carotenoid level not accounted for by the model (assumed normally distributed). We will additionally include a random intercept, $\alpha ,$ for each subject in the study to account for within-subject correlation. The primary assessment of the effect of early disclosure will compare the adjusted mean carotenoid level between high-risk subjects assigned to early disclosure (${\beta }_{6t})$ and all subjects assigned to late disclosure, irrespective of AMD risk ($1/3({\beta }_{3t}+{\beta }_{5t}+{\beta }_{7t}))$, with $t=12$ months. Statistical significance will be set at 2-tailed *p* < 0.05 for all analyses.

AMD risk alleles will be individually tested for associations with skin carotenoid status and MPOV using simple linear regression. We will use this step as a variable selection for a multivariable regression. Alleles with *p*-values less than 0.05 in the univariable analyses will be included in the final multivariable regression, which will additionally control for baseline measures of ocular and systemic carotenoid status.

### Interim analyses {21b}

Not applicable; there will be no interim analysis in the MAGENTA study.

### Methods for additional analyses (e.g., subgroup analyses) {20b}

Subgroup analyses will be performed to assess the impact of AMD genetic risk disclosure on participant dietary carotenoid intake, co-existing medical conditions, BMI, and other factors on our study results, with proper attention to the effects of multiple tests on the ability to draw a conclusion.

### Methods in analysis to handle protocol non-adherence and any statistical methods to handle missing data {20c}

To assess the risk of bias due to missing data, patterns of missing measurements across follow-up visits will be displayed for the primary and secondary outcomes. Reasons for missing measurements will be tabulated with a focus on differentiating missed measurements for logistical reasons from dropout or intermittent missingness potentially related to the condition of the participant.

Because we are using restricted maximum likelihood estimation, our statistical inferences for the primary and the numeric secondary outcomes will remain valid if missing data follows a missing at random (MAR) pattern [49]. Nonetheless, if more than 10% of subjects have missing outcome measurements for any of the follow-up visits, i.e., we have fewer than 72 complete subjects, or if comparisons of baseline characteristics between subjects with missing and non-missing measurements indicate significant imbalances, we will apply multiple imputations (MI) to further address missing outcome scores [50]. MI incorporates baseline and follow-up factors beyond the variable being analyzed into imputation models to account for the dependence of missing data on other factors. We will use the fully sequential imputation method to generate imputed values [51].

### Plans to give access to the full protocol, participant level-data and statistical code {31c}

The protocol of the study is publicly available on ClinicalTrials.gov identifier (NCT05265624). The principal investigator will give access to the study’s de-identified dataset upon reasonable request.

## Oversight and monitoring

### Composition of the coordinating center and trial steering committee {5d}

As earlier indicated, this study is a single-site randomized controlled trial, and as such, the co-PIs (PSB and MEH) and the study team at the John A. Moran Eye Center will manage the trial. The trial steering committee, consisting of the data and safety monitoring committee (DSMC), co-PIs, and regulation committee, will ensure the accuracy and completeness of the study data. Notably, the committee will have overall responsibility and authority for directing activities, formulating policies for the study, and changing the study protocol.

### Composition of the data and safety monitoring committee, its role and reporting structure {21a}

The DSMC members will include the co-PIs, an independent ophthalmologist, and a licensed clinical social worker. The selection of DSMC members will be based on clinical expertise, knowledge of clinical research methodology and regulations, and the absence of conflicts of interest. The DSMC will meet at least every 6 months (or as deemed appropriate by the chairperson) at a suitable and convenient time for all members. The DSMC will assist the sponsor-investigator in protecting the interests of study participants and assuring the integrity of study conduct and results.

### Adverse events (AEs) reporting and harms {22}

The study investigates behavioral changes following AMD genetic risk disclosure, so few, if any, medical adverse effects are expected with the study intervention. However, any unfavorable and unintended sign, including a clinically significant abnormal laboratory finding, symptom, or disease temporally associated with study allocation will be monitored and reported in detail in the patient source documents from the signing of the ICF until the final visit. The co-PIs will evaluate and treat/follow up with all AEs until symptoms or values return to normal. Further, the various regulatory bodies (IRB and FDA) will be informed of the various AEs and appropriate measures executed accordingly.

### Frequency and plans for auditing trial conduct {23}

The regulatory coordinator and auditor from the ethics committee of the University of Utah will have monitoring visits every 3 months. The monitoring team will review the study conduct and compliance with the study protocol, good clinical practice, standard operation procedures, and applicable regulatory requirements.

### Plans for communicating important protocol amendments to relevant parties (e.g., trial participants, ethical committees) {25}

Any amendments to the study protocol deemed necessary will be discussed between the co-PIs and the DSMC. The investigator will not implement any changes to the protocol without prior review and documented approval from the IRB of an amendment, except where necessary to eliminate immediate hazards to study subjects.

### Dissemination plans {31a}

The study findings will be disseminated through peer-reviewed scientific journal publications and conference presentations. Moreover, study results will be communicated at relevant professional, clinical, and scientific meetings.

## Discussion

The MAGENTA study is the first prospective trial designed to test whether disclosure of a high genetic risk of progression to advanced AMD will promote sustained (i.e., 1 year), quantifiable long-term changes in behavior and lifestyle associated with lower incidence of AMD later in life in clinically healthy individuals. The AAO, based on its expert panel opinion, discourages genetic testing for AMD since there is no evidence of genetic knowledge changing the disease course [9, 10]. Because of the paucity of research data to support the AAO’s position on AMD genetic testing, the expert panel specifically suggested that such clinical studies should be conducted in the future. Therefore, the MAGENTA trial will provide relevant empirical evidence to either increase the utilization of genetic testing to identify individuals at high genetic risk of AMD so that they can initiate lifestyle changes long before signs and symptoms appear or provide additional support to continue AAO’s current stance against pre-symptomatic testing for AMD genetic risk. Moreover, findings from MAGENTA will serve as pilot data to design future larger-scale, longer-term, multicenter phase 3 trials.

A novel and important characteristic of the MAGENTA trial worth highlighting is the use of skin carotenoid measurement (resonance Raman spectroscopy (RRS) and reflectance spectroscopy (RS)) and imaging of macular pigment with the Heidelberg Spectralis (autofluorescence imaging (AFI) and fluorescence lifetime imaging ophthalmoscopy (FLIO)) as biomarkers of healthy lifestyle choices in response to a disclosure of genetic risk for a degenerative disease such as AMD. This has never been conducted systematically in the past.

Despite our study advancing our knowledge and understanding of genetic testing and its impacts on pre-symptomatic individuals, it is important to note that we will not assess participants’ visual performance. This is because the study is designed to primarily ascertain changes in systemic and ocular carotenoids as a biomarker for healthy lifestyle upon knowledge of AMD genetic risk. Also, we are cognizant of other healthy lifestyle choices such as limited exposure to sunlight and increase in physical activity impacting carotenoid levels. We plan to feature these expanded measures of lifestyle modifications and potential active nutritional interventions in future trials.

MAGENTA trial will have considerable public health benefits, as it will provide relevant findings that may reduce the financial and social burden of AMD and allow individuals to implement strategies that can prevent or delay the onset of the disease.

## Trial status

The MAGENTA trial is conducted using protocol version IRB_00150093 approved on February 8, 2023. Trial recruitment began on August 3, 2022, and it is still open for enrollment. Presently, we are 75% (60/80) enrolled, and we expect to finish recruitment in September 2023.

## Data Availability

Data will be available upon reasonable request from the Principal Investigator.

## Abbreviations

AAO: American Academy of Ophthalmology
AFI: Autofluorescence imaging
AMD: Age-related macular degeneration
ANSI: American National Standards Institute
AREDS: Age-related Eye Disease Study
ARMS2: Age-related maculopathy susceptibility protein 2
BMI: Body mass index
CCTS: Center for Clinical and Translational Science
CFH: Complement factor H
CFHR5: Complement factor H-related 5
CRF: Case report form
DSMC: Data Safety and Monitoring Committee
FAF: Fundus autofluorescence
FDA: Food and Drug Administration
FLIO: Fluorescence lifetime imaging ophthalmoscopy
HADS: Hospital Anxiety and Depression Scale
HIPAA: Health Insurance Portability and Accountability Act
HPLC: High-performance liquid chromatography
HTRA1: High-temperature requirement factor A1
ICF: Informed consent form
ICH: International Conference of Harmonization
IES: Impact of Events Scale
IR: Infrared reflectance
IRB: Institutional review board
L: Lutein
LSC: Long spectral channel
LZQ: Lutein Zeaxanthin Questionnaire
MAGENTA: Moran AMD Genetic Testing Assessment Study
MAR: Missing at random
MI: Multiple imputation
MP: Macular pigment
MPOD: Macular pigment optical density
MPOV: Macular pigment optical volume
MZ: *meso**-*Zeaxanthin
NHANE: National Health and Nutrition Examination Survey
PI: Principal investigator
REDCap: Research Electronic Data Capture
RRS: Resonance Raman spectroscopy
RS: Reflectance spectroscopy
SPIRIT: Standard Protocol Items Recommendations for Interventional Trials
SSC: Short spectral channel
Z: Zeaxanthin
